# New Tools to Support the Risk Assessment Process of Nanomaterials in the Insurance Sector

**DOI:** 10.3390/ijerph18136985

**Published:** 2021-06-29

**Authors:** Francisco Aznar Mollá, Carlos Fito-López, Jose Antonio Heredia Alvaro, Francisco Huertas-López

**Affiliations:** 1 Research Line Sustainable Chemistry and Supramolecular Chemistry, Universitat Jaume I, 12071 Castelló, Spain; al363139@uji.es; 2 ITENE, Technological Institute of Packaging, Transport and Logistics, 46980 Paterna, Spain; maida.domat@itene.com; 3 Industry 4.0 Chair Director, Universitat Jaume I, 12071 Castelló, Spain; heredia@uji.es

**Keywords:** nanotechnology, nanomaterial, nanoparticle, ecotoxicity, risk assessment, modeling

## Abstract

During the last decade, the use of nanomaterials, due to their multiple utilities, has exponentially increased. Nanomaterials have unique properties such as a larger specific surface area and surface activity, which may result in health and environmental hazards different from those demonstrated by the same materials in bulk form. Besides, due to their small size, they can easily penetrate through the environmental and biological barriers. In terms of exposure potential, the vast majority of studies are focused on workplace areas, where inhalation is the most common route of exposure. The main route of entry into the environment is due to indirect emissions of nanomaterials from industrial settings, as well as uncontrollable releases into the environment during the use, recycling and disposal of nano-enabled products. Accidental spills during production or later transport of nanomaterials and release from wear and tear of materials containing nanomaterials may lead to potential exposure. In this sense, a proper understanding of all significant risks due to the exposure to nanomaterials that might result in a liability claim has been proved to be necessary. In this paper, the utility of an application for smartphones developed for the insurance sector has been validated as a solution for the analysis and evaluation of the emerging risk of the application of nanotechnology in the market. Different exposure scenarios for nanomaterials have been simulated with this application. The results obtained have been compared with real scenarios, corroborating that the use of novel tools can be used by companies that offer risk management in the form of insurance contracts.

## 1. Introduction

The main principle of insurance is that one party, identified as “the insurer,” shall guarantee payment for an uncertain future event related to an incident where potential damages can be expected on different subjects, including human health or the environment. Special types of insurance policies that insure against specific types of risks faced by a particular company are required. In this regard, a nanotechnology-based company needs a policy that covers damage or injury that occurs as a result of an accidental event in the factory, including accidents involving nanomaterials.

Today, engineered nanomaterials (ENMs) are frequently used. Their applications range from scratch-resistant or self-cleaning surface coatings to enhanced cosmetics. Applications in food include objectives to enhance flavor and texture and encapsulate micronutrients to prolong their stability, augmented by packaging applications to prolong shelf life and avoid bacterial contamination. Potential applications in other sectors include environmental remediation to detect and eliminate toxic substances, energy generation and storage plus multiple other commercial uses of novel materials [1]. Besides the wide range of new opportunities offered by these novel materials, concerns have been raised because of potential adverse health effects that may arise if NMs are taken up by the human body [2].

While human exposure to NMs may in principle occur during any stage of the material’s life cycle, it is most likely in workplaces, where these materials are handled or produced in large quantities or over long periods of time. Inhalation is considered the most critical uptake route as these small particles are able to penetrate deep into the lung and deposit in the gas exchange region. Inhalation exposure to airborne nanomaterials, therefore, needs to be assessed in view of worker protection. However, to date, unlike what happens for gaseous compounds, there is still no methodology clearly established by the scientific community to evaluate the exposure of nanomaterials in the workplace.

The majority of the literature regarding the fate and transformation processes of NMs in the environment focuses on aquatic systems and soils (e.g., Baalousha et al. [3]; Gutleb et al. [4]), since the major part of NMs is known to end up in these two compartments, whereas only 0.1–1.5% of the produced NMs are estimated to be released into the atmosphere during their life cycle [5]. However, since inhalation has been identified as the main penetration route of exposure for human beings, monitoring the presence of NMs in air, especially in workplaces, is crucial.

Many activities involved in engineered nanomaterial (ENM) manufacturing may be a potential source of ENM emissions. In many cases, the major emissions come from the process step generating the nanomaterial, but at a subsequent phase, such as recovering particles from the reactor, milling, drying or further handling may also be a potential NM source [6]. Other processes such as spray drying or milling are more prone to leaks as they are often performed in air at atmospheric pressure conditions [7]. Adopting the corresponding safety measures throughout the NM life cycle can minimize and even avoid the exposure of workers to nanomaterials. Nevertheless, while systematic efforts are made to prevent them, accidental releases may generally still occur in the chemical industry. Major releases are very rare events, but if they occur, they can contribute significantly to the emission of chemicals to the environment and can be a serious hazard to workers if proper precautionary measures are not taken (e.g., personal protective equipment). At each stage of the ENM life cycle, accident scenarios can take place that lead to unintended or uncontrolled releases of ENMs to different environmental compartments: air, water or soil.

Release rates and amounts are very dependent on the release scenario, the safety procedure in place, and the location and environment of the release [8]. They can range from a few kilograms for small releases to several tons in rare cases of major releases. Under an accidental scenario the release form is directly related to the form of ENMs used in the industrial process. Nanoparticles typically agglomerate with agglomerate sizes in the micrometric range.

Several uncontrolled releases involving ENMs have occurred in the past. Some examples can be found in the ARIA database [9]. These accidents had no reported health or environmental consequences but resulted in the release of measurable amounts of ENMs to the immediate surroundings.

Small-scale accidents related to the handle nanomaterials have also been reported in literature, such as leaks from an inadequately sealed vacuum cleaner (Boowok et al. [10]) or the miss function of bag filters (Jin et al. [11]).

To date, there is still a lack of information regarding the threshold limits for occupational exposure. International organizations such as NIOSH have established recommended exposure limits (RELs) for a limited type of particles such as ultrafine TiO_2_, CNT and nanofibers [12,13]. To date, different approaches have been adopted to assess the risk of NM exposure in the workplace. The ideal option would be to carry out a quantitative evaluation by carrying out experimental measurements in the work environment itself. However, this option is not always possible, so qualitative methods are commonly used. These methods can be used to make a first approximation or diagnosis of the hygienic situation derived from the presence of chemical agents and of the necessary preventive measures in each case. Some of the most recognized and used qualitative evaluation methods for nanomaterials are the CB Nanotool method developed by Zalk et al. [14], applicable for small amounts of NMs, in laboratories or small-scale production, Stoffenmanager Nano (http://nano.stoffenmanager.nl./, accessed on 21 October 2020 [15]) and the ISO/TS 12901-2:2014 method (http://iso.org/ accessed on 17 September 2020 [16]), which are only applicable for research laboratories and industrial scale to assess the inhalation risk of particles with water solubility <0.1 g/L, individual particles, aggregates and agglomerates. Although these methods may be useful, their limitations are crucial.

Moreover, the nanotechnology-related industry requires insurance to efficiently manage risks that arise from running the business, considering the current significant knowledge gaps for nanotechnology risk assessment [17,18,19]. Several tools are already available for risk assessment, including the Swiss Precautionary Matrix [20,21], NanoRiskCat [22,23], the US Control Banding Nanotool [24] or NanoSafer [25]. However, these models and tools require extensive expertise and knowledge of nano-safety and were made for different purposes and application domains (i.e., inhalation, dermal, sprays, etc.), making them inappropriate for non-experts. Concerning risk management and insurance, a limited number of tools are currently available, including CENARIOS [26], LICARA NanoScan [27] or the IRGC framework [28]. A list of the tools available for risk assessment is shown in Appendix A.

The aim of this study is to present a simple application for the risk assessment of ENMs in the insurance sector, developed by integrating hazard-related data retrieved from the eNanoMapper database [29], and optimized exposure models developed under the SUDOE project NanoDESK [30,31,32,33]. These models aimed to evaluate the levels of occupational exposure to nanomaterials, their aggregates and/or agglomerates (NOAA) during the manufacture of polymer nanocomposites, end-of-life processes and/or their use in consumer articles, and the estimation of unintentional emissions of nanomaterials into the environment. Specifically, the present work focuses on (1) the characterization of aerosol particles released under different scenarios, and (2) to assess the potential use of the tool by comparing estimated data with measured data. An operative version of the tool can be downloaded from the URL: https://www.cyc-ingenieros.com/nanoserpa/ (accessed on 25 February 2011).

## 2. Methodology

### 2.1. Development of the NanoSerpa Application

**Figure 1:**
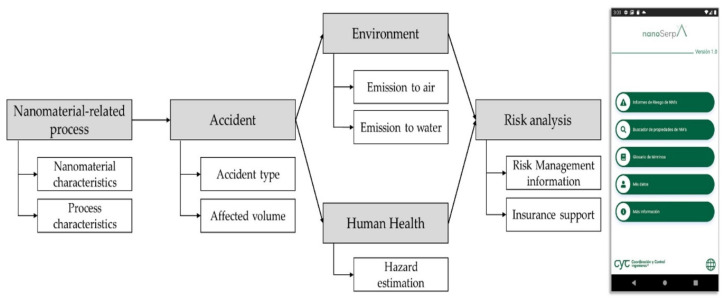
NanoSerpa application flow diagram (**left**) and screenshot of the app (**right**).

The application was designed to be used as a library to search and consult the properties of existing nanomaterials and to easily elaborate accident reports where nanomaterials are involved. For the latter, certain input parameters are required from the user: (1) type of nanomaterial; (2) amount of nanomaterials involved in the accident; (3) process that was taking place (synthesis, manufacturing, etc.); (4) type of accident (fire, explosion, etc.); (5) optional comments about the accident. Once all fields are completed, a series of probabilistic models and auxiliary tables will be executed resulting in emission, health hazard values and risk indices. These models have been created using the open software Python [34], and their functionalities are briefly explained below. Figure 1 depicts a flow diagram of the application.

- Release and emissions to the environment: A material flow model (MFM) has been implemented for the estimation of emissions into the environment. This model has included the most common processes when working with nanomaterials: synthesis, manufacturing, use, sludge treatment disposal, incineration removal and filtration. In addition, different types of accidents such as explosions, burning or fire, gas escapes and spillovers have been introduced.
- Health hazard: This model considers 10 different endpoints when analyzing the health hazards of nanomaterials. This information is available in the NanoSerpa database, which contains information from different data sources, highlighting the eNanoMapper database [29]. eNanoMapper is the biggest European public database hosting nanomaterial characterization data and biological and toxicological information.
- Risk index: The risk index is estimated based on the two models mentioned above. This type of band model is widely used in the field of nanoparticle risk calculation.

#### 2.1.1. Emission/Release Estimation Model

To estimate the exposure potential, the model considers relevant determinants of exposure based on Scheneider et al. [35]. The relevant determinants considered were energy and duration of the process/activity, volume of the facility (personal area, room, industrial facility or surrounding area), dustiness (very high (extremely fine and light powder), high (fine powder), medium (coarse powder), low and very low (extremely coarse powder)), solubility, weight fraction (% purity), moisture level, viscosity, and amount used. The NanoSerpa model was developed based on Michael P. Tsang [36] and Ganser et al. [37] and adapted to require the minimum user input as possible, as this was one of the biggest concerns for developing the app.

The concentration of nanomaterial released in μg/m^3^ in the affected zone by the accident is calculated using the equation below:

(1) $$C\;\left(\frac{\mu g}{m^{3}}\right)=\frac{M_{\mathrm{NM}}{*\mathrm{EHP}*K}_{\mathrm{NM}}*A}{V}$$

where C is the released nanomaterial concentration in μg/m^3^, M_NM_ is the mass of the nanomaterial in μg, V is the facility/room volume in m^3^, and EHP, K_NM_ and A are pre-calculated ponderations based on the energy handling potential, physicochemical properties, which are state dependent (viscosity/solubility for liquids, dustiness/moisture for solids), and the liberation coefficient for the accident, respectively.

Then, a probabilistic nanomaterial flow analysis model based on the transfer coefficients (TCs) proposed by Gottschalk [38] and Spinazze [39] was implemented into the NanoSerpa tool to estimate the different amounts of nanomaterials potentially released due to the manufacturing or usage of nanoparticle-containing products. This mass-balanced-model allows treating all parameters as probability distributions. Therefore, the model output is represented by ENM flow probability distributions. Model input and output distributions were derived by the Monte Carlo method and implemented in the Python code. After running the model, the final emissions to air, water and soil sediment are calculated (as µg/m^3^) using assumed transfer coefficients shown in Table 1.

Uncertainties were taken into account for the input ENP production value and for the TCs, working with distributions. To facilitate the interpretation of results, emission levels have been established based on the extrapolation of the PM2.5 fraction to the US EPA air quality index [40]. For an emission value below 13 μg/m^3^, a very low level is established; for a value between 13 and 25 μg/m^3^, a low level is established; for a value between 25 and 45 μg/m^3^, a medium level is established; for a value between 45 and 56 μg/m^3^, a high level is established; and for a value above 56 μg/m^3^, a very high level is established.

#### 2.1.2. Hazard Estimation Model

The hazard estimation model is able to find in the NanoSerpa database up to 10 different endpoints, including 3 physicochemical properties that directly or indirectly affect the potential hazard of the nanomaterials following a structure–property–hazard (SPH) relationship [35] (particle size, *Z*-potential and aspect ratio) and 7 key toxicological endpoints (EC20, EC50, LC20, LC50, % viable cells, cell cycle and genotoxicity/DNA in tail). This database has been built and adapted from different data sources, highlighting eNanoMapper [29] as the biggest nanomaterial database, hosting hundreds of nanomaterial properties. The NanoSerpa database is designed to be updated when new data become available.

For the estimation of the hazard, each endpoint is analyzed independently and evaluated from very low to very high hazard. The worst-case approach is followed in this model, so if more than one endpoint is found in the database, the worst value is taken for estimating the risk.

#### 2.1.3. Risk Index Characterization

Both results (emission and hazard) are taken into account to estimate the potential risk index. Table 2 represents how this index is calculated from the results of the hazard calculation and emission index.

### 2.2. NanoSerpa Case Studies

To validate the use of the NanoSerpa application, four events reported in the literature have been simulated:

- SiO_2_ nanoparticle leak from a vacuum cleaner
- Release of TiO_2_ nanopowder from a bag filter system
- Fall of eight TiO_2_ bulk bags on the road
- Leak of black carbon during transportation

Besides, two real cases involving ENMs were analyzed at ITENE facilities:

- Spillage of paint containing graphene during spraying
- Accidental spillage of a dry mortar

In these studies, exposure measurements were carried out applying tier 2 and tier 3 approaches to gather information, considering a suit of instruments to monitor the levels of exposure, including a particle counter (CPC—TSI Model 3007) an optical particle sizer (OPS—TSI Model 3330), which provides data on particle size distributions, as well as the NanoScan SMPS Model 3910, which provides measures of the particle size distribution. Technical personnel, trained in the use of these instruments, recorded all the events that occurred during the entire duration of workplace monitoring in a specific event log.

The inlets of the devices located in the near field (NF) were approximately at a height of 1.5 ± 0.1 m and ~0.5 m from the worker. The exposure was assessed by measuring directly in the personal breathing zone (PBZ) of the operator, defined as a 30 cm hemisphere around the mouth and the nose (EN, 2012). Flexible 80 cm Tygon^®^ tubes were attached to the inlets of the instruments (CPC 3007, TSI NanoScan, and OPS 3330) to achieve the worker’s breathing zone. The far-field (FF) devices (OPS/CPC) were placed from 6 to 12 m.

The direct reading measurement instruments were complemented with filter-based air samples (37 mm cassettes) collected during the sampling campaign for morphological and compositional data of airborne and settled particles, respectively. These air samples were collected from the breathing zone using an APEX (Casella CEL) personal sampling pump at a flow rate of 3.5 L min^−1^ and a polycarbonate filter. The samples from the far field locations were also collected at a 1.5 m height. The filters collected were further analyzed by scanning electron microscopy/energy-dispersive X-ray spectroscopy (SEM/EDXS).

The data retrieved from the real exposure scenarios were statistically analyzed to determine the arithmetic mean and maximum and minimum number concentrations in each exposure scenario. Mass concentrations were obtained directly from the OPS.

The background levels were established considering the data on the ENM concentration retrieved before the operations involving ENM began. Comparison of background levels with measured concentrations (taken when the process is in operation) was carried out to identify any increases in the levels. Any enhanced concentration levels were then assigned to emission sources or activities using the activity/time log. The morphology and chemical nature of the ENMs retained in the polycarbonate filter were used to “speciate” the real-time quantitative measurements in order to distinguish ENM from incidental nanoparticles in the workplace.

## 3. Results and Discussion

### 3.1. Accidental Spillage of Paint Containing Graphene during Low-Density Paint Spraying

Experimental data have been collected at the ITENE pilot plant during the application of acrylic containing 0.1% m-GO graphene. Acrylic paint was sprayed using a gravity spray gun powered by a 0.65-L glass, capable of working at a maximum air inlet pressure of 8 bar (116 psi), when the contents of the boat were spilled when filling the gun. The operator wore a double nitrile glove, a Tyvek suit and a full mask with an FFP3 filter.

Possible exposure to graphene during paint spraying was measured using a condensation particle counter (CPC—TSI-3007) and an optical particle sizer (OPS—TSI-3330). Measurements of particles in the environment were conducted, so possible air exposure to graphene was recorded during the incident.

Table 3 shows the concentration values recorded by the CPC and OPS that day. The results are weighted to the graphene content of the mixture. The RCR, obtained (Equation (2)) as the quotient between the concentration of the personal or workplace area and a chemical reference toxicity value, is significantly lower than 1, which implies a very low risk possibility. In this case, the mean toxicity values employed are predicted non-effect concentration (PNEC) = 9.37 × 10^4^ particles/cm^3^ and derived non-effect limit (DNEL) = 0.0446 mg/m^3^, calculated with SECO DNEL Tool [41].

(2) $${RCR}_{average}=\frac{Mean\;Exposure}{Mean\;Toxicity}$$

**Figure 2:**
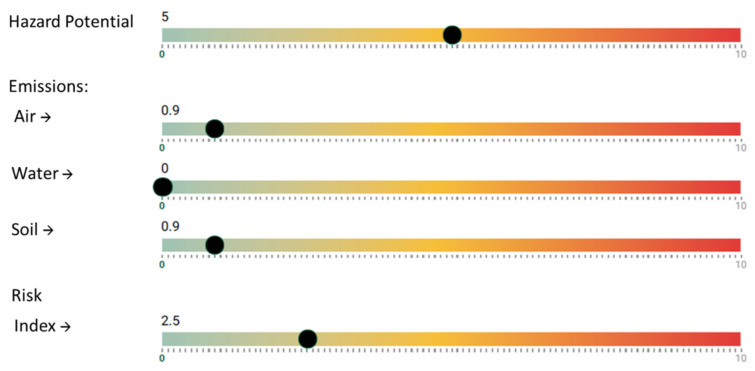
Excerpt from the report generated with the NanoSerpa v1.0 app for a spillage of paint containing graphene during spraying. These results apply only to the exposure area where the events studied have occurred.

By applying this information to the NanoSerpa v1.0 app, we obtain the report that has been summarized in Figure 2. The input data used to run the study is shown in Table 4.

In Figure 2, it is observed that the risk of air exposure, which is the main penetration route for human exposure, is very low (<1). In this case, the dermal risk would be the most likely. However, when wearing a protective suit Category III, the worker would be well protected for this unexpected event. The estimated emissions to the air and soil would be very low (<1) and non-existent in the case of water emissions.

The risk index obtained with NanoSerpa v1.0 is 2.5, which means a low risk, considering also a very low air emission. The results obtained by the apps agree with the values of RCR experimentally obtained, which also suggest a low risk.

### 3.2. Accidental Spillage of a Dry Mortar

The overall average particle number concentration measured with a TSI condensation particle counter (CPC 3007) during the activity period (3.1 × 10^4^ particles/cm^3^) was significantly above the background level (9.6 × 10^3^ particles/cm^3^). The data from the activity showed several peaks with concentrations up to 3.5 × 10^4^ particles/cm^3^, more than 3 times the concentration found in the background. Such a change between activity and background coincided with the accidental spillage of the 25 kg plastic-lined paper bag.

**Figure 3:**
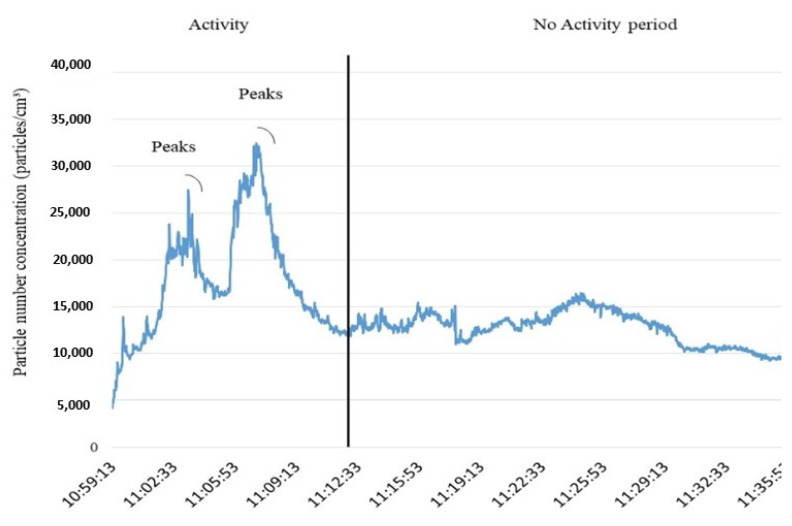
Concentration of particles measured with a CPC during the activity (**left**) and the background (**right**).

Figure 3 shows the variations in the particle number concentration measured in the PBZ during the operation, with a sharp increase immediately after the accidental spillage of the photocatalytic cement paper bags containing TiO_2_ nanoparticles. The highest peak values obtained for this activity were 3.5, 3.1 and 2.7 (×10^4^) particles/cm^3^, which are about 3 and 2.5 times higher than the background levels.

**Figure 4:**
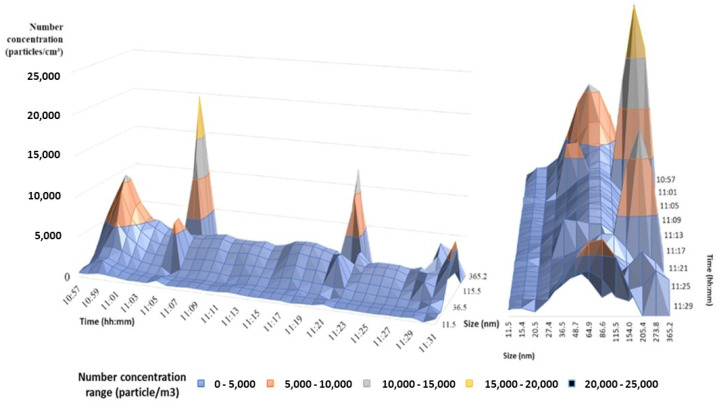
Concentration of particles measured with a NanoScan during an accidental spillage of photocatalytic cement paper bags containing TiO_2_ nanoparticles.

The analysis of the data measured by the nanoparticle sizer (TSI NanoScan 3910) showed an average particle size of ~83 ± 2 nm. The maximum peaks observed with the CPC were also observed with the SMPS, being mainly due to an increase in the number of particles ranging from 115 to 360 nm, as can be derived from the 3D picture depicted in Figure 4. This figure shows two main modes corresponding to particles with an average particle size of ~71 ± 2 nm and ~237 ± 2 nm, respectively.

Table 5 shows the overall averages of PM10, PM2.5, PM4 and PM1 during accidental spillage. These PM fractions were found to be significantly above the non-activity levels. All fractions were up to 10 times larger during the accident than during subsequent periods of non-activity.

**Figure 5:**
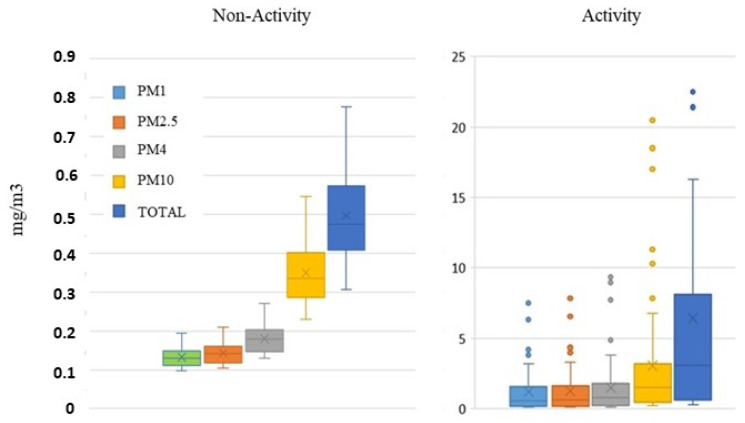
Boxplot of PM levels during the accidental event and non-activity periods.

Figure 5 shows a boxplot of the PM fractions analyzed during this event. The data depicted in the figure reinforce the idea that an accidental spillage is able to release particles into the workplace, including both particles in the nanometer range, as derived from the CPC and SMPS, as well as large particles, as measured by the SMPS.

As can be seen from the experimental data reported, the levels of particles measured with the CPC and the Nanoscan device are considerably higher than concentrations during non-activity periods. The RCR, calculated from the quotient of the total concentration (Table 5) and the threshold limit value (10 mg/m^3^ for TiO_2_, [42]), is 0.64, which implies a low risk possibility.

**Figure 6:**
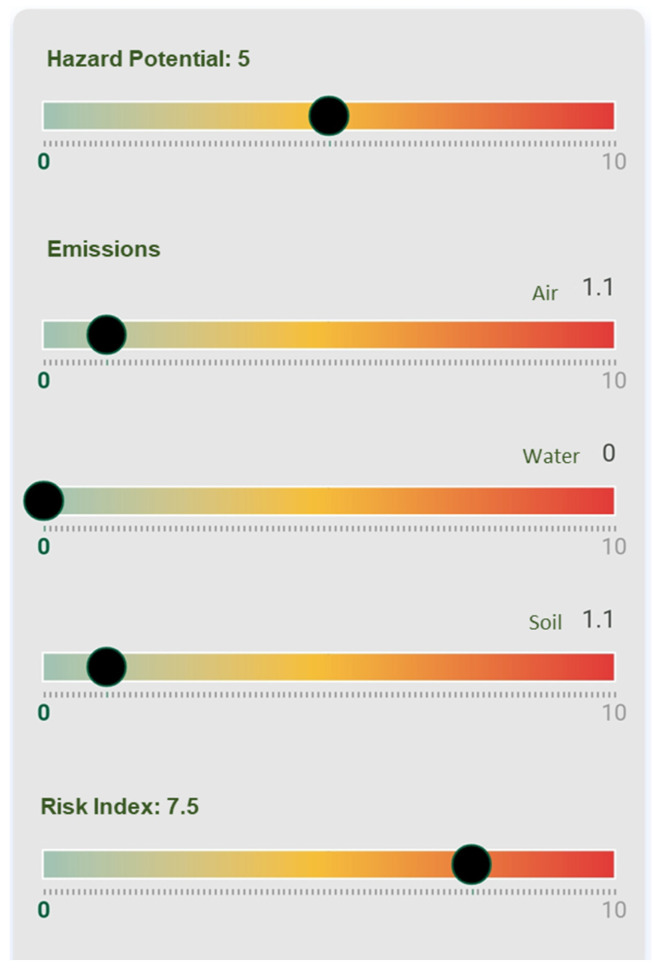
Excerpt from the report generated with the NanoSerpa v1.0 app for an accidental spillage of a dry mortar containing TiO_2_. These results apply only to the exposure area where the events studied have occurred.

As in the previous case, by applying this information to the NanoSerpa v1.0 app, we obtain the report that has been summarized in Figure 6. The input data used to run the study are shown in Table 6.

The main penetration route for human exposure during the spillage is inhalation and dermal absorption. In Figure 6, it can be seen that the risk of air exposure is low (1.1 on a scale of 0–10). The same results have been obtained from the experimental risk evaluation. In this case, since the dermal risk could also be important, the use of a protective suit Category III could also be considered for the worker in order to be protected for this unexpected event.

Moreover, although the air emission risk is low, high peak concentrations were measured, leading to concentrations even 10 times higher than non-activity periods in the case of total particle concentration, summarized in Table 5. This fact is in agreement with the medium–high risk index (7.5) estimated with the NanoSerpa app.

This new app also proposed, considering the hazard potential, a list of preventive/corrective actions that could be taken. For a scale of 2–5, which is the case with this spillage and the rest of the accidental scenarios considered in this work, the suggested actions are as follows:

1. Implementation of engineering controls, including forced ventilation and/or containment systems.
2. Use of individual protective equipment according to the route of exposure.
3. Any technical assistance that, due to the characteristics of the situation and the material, the inspector technician considers necessary to apply.

The estimated emissions to soil would be low (1.1) and non-existent in the case of water emissions. However, since the spillage took place in a closed area with a paved ground, soil emissions are not relevant.

A comparison of the risk characterization ratio (RCR) calculated using experimental data obtained in these two real case scenarios with the risk index and air emissions reported by the NanoSerpa app is shown in Table 7. Information about the risk scale employed in the NanoSerpa app has previously been shown in Table 2.

As can be seen in Table 7, a comparison between the air emission risk (NanoSerpa) versus the risk calculated from real measurements carried out in the workplace has been done. The same comparison can be done for the risk index and personal RCR. The results obtained are, in both cases, in good agreement.

### 3.3. Literature-Based Scenarios

For the following cases, the experimental data found are not as detailed as in the previous case; therefore, a qualitative comparison has been made. The input data required to run NanoSerpa for each scenario are depicted in Table 8.

#### 3.3.1. SiO_2_ Nanoparticle Leak from a Vacuum Cleaner

The following case proposed is the SiO_2_ nanoparticle leak from a vacuum cleaner (Boowook et al. [10]). In this case, the workers were exposed to the high concentration of nano-silica emitted into the air when poured into a container or when moving the container. It was found that the use of a vacuum cleaner with a leak caused by an inadequate seal was the source of the nano-silica dispersion in the inner air.

The study concluded that there was a risk of the leakage of these particles during vacuuming. Although the size of the nano-silica particles that were emitted into the air (during the handling of the nano-silica by a worker) was mostly greater than 100 nm or several microns (µm) due to the coagulation of the particles, those that filtered from the vacuum cleaner were similar in size to that of the primary particle (11.5 nm). Nanoparticles were generated also during the operation of the filter press and ultrasonic cleaning, but they were oil particles and water particles, respectively (Boowook et al. [12]).

**Figure 7:**
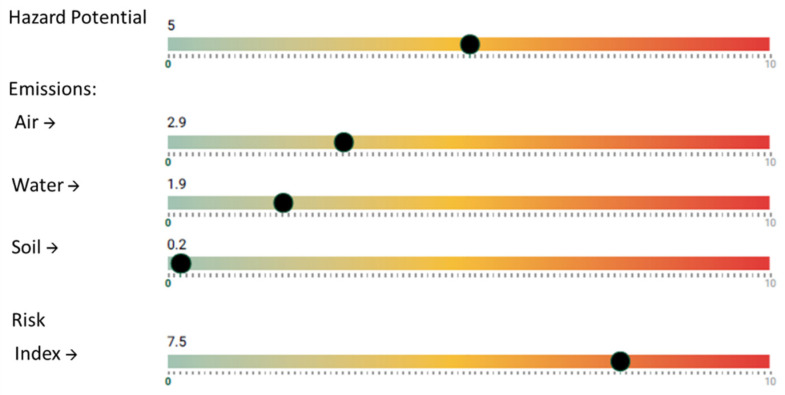
Excerpt from the report generated with the NanoSerpa v1.0 app for a SiO_2_ nanoparticle leak from a vacuum cleaner. These results apply only to the exposure area where the events studied have occurred.

Emissions simulated by the NanoSerpa v1.0 app to water and soil are 1.9 and 0.2, respectively. In this case, the results of the NanoSerpa v1.0 app show a higher risk to inhaled health (risk index 7.5 on a scale of 0–10; see Figure 7). Although there is clear exposure to escaped nanomaterials (air emission 2.9), this exposure is very brief and punctual. The authors conclude that high-concentration nanoparticles are emitted to the air while pouring and transferring nano-silica. Therefore, a respirator capable of capturing nanoparticles must be worn, and activities must be carried out within the HEPA-filtered hood. A regular check on the vacuum cleaner is necessary to prevent leakage of nanoparticles. Additionally, wet cleaning is safer in reducing exposure risk (Boowook et al. [10]).

#### 3.3.2. Release of TiO_2_ Nanopowder from a Bag Filter System

The second real case considered was the release of TiO_2_ nanopowder from a bag filter system (Ji et al. [13]). This study detected the presence of nanoaerosols in a laboratory used to manufacture titanium dioxide. TiO_2_ nanopowder was produced by flame synthesis and collected using a bag filter system for subsequent harvesting. However, it was shown that the particle collection efficiency of the bag filter system was only 20% for a particle diameter of 100 nm, which is much lower than the performance of a high-efficiency particle air filter (HEPA). In addition, the laboratory hood system was inadequate to completely renew the discharged air from the bag filter system. The imbalance in airflow speeds between bag filter and laboratory hood systems could lead to high exposure to nanopowder in laboratory environments, putting the long-term integrity of workers at risk (Ji et al. [13]).

**Figure 8:**
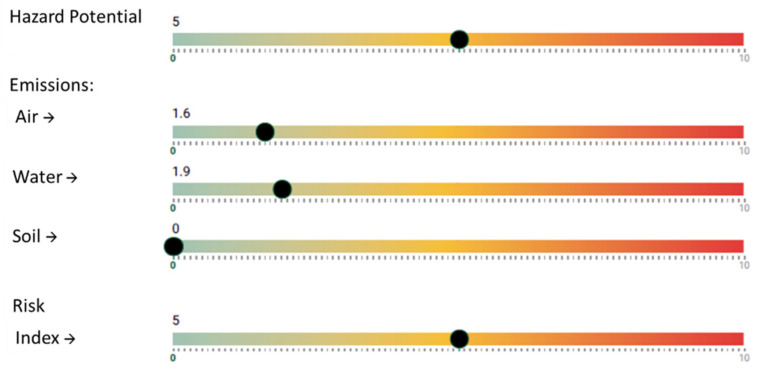
Excerpt from the report generated with the NanoSerpa v1.0 app for the release of TiO_2_ nanopowder from a bag filter system. These results apply only to the exposure area where the events studied have occurred.

By entering this information in the NanoSerpa v1.0 app, a report like the one shown in Figure 8 is obtained. It shows some risk of air emissions (1.6 points), as filtration systems are trusted and they are not fulfilling their role properly. Despite the personal protection systems present (FFP3 filter mask, gloves, gowns and universal mounted goggles), engineering systems should be checked to improve on-site ventilation.

#### 3.3.3. Leak of Black Carbon during Transportation

**Figure 9:**
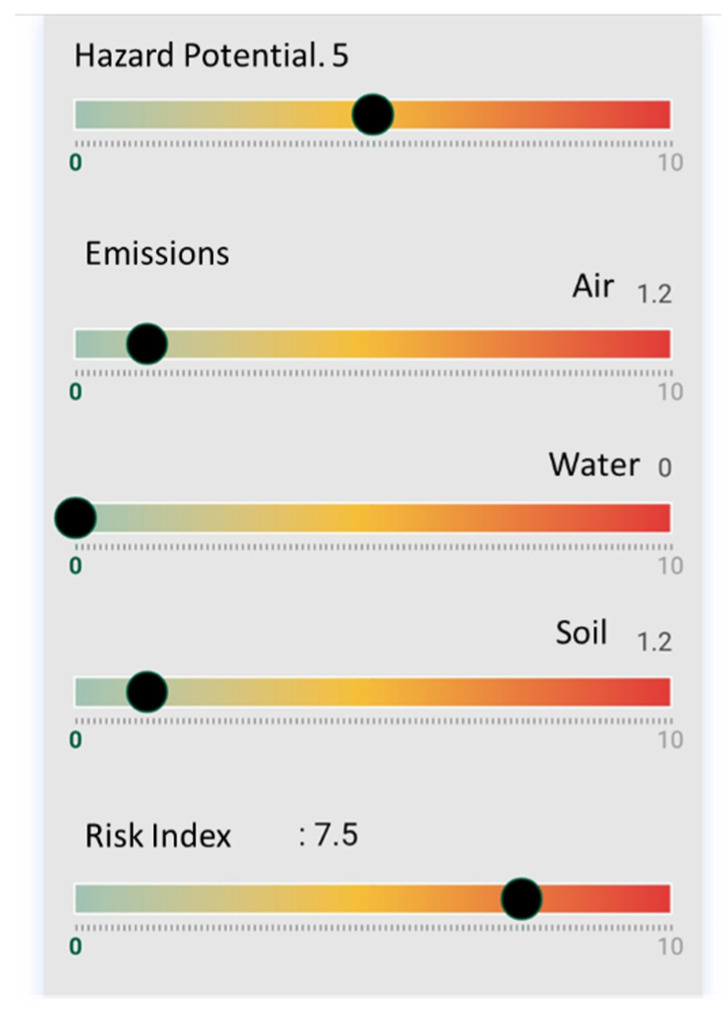
Excerpt from the report generated with the NanoSerpa v1.0 app for a leak of black carbon from a pneumatic transport pipe.

A leak from a pneumatic transport pipe (Blanzy, France in 2012) of about 5 tons of carbon black was studied. By entering the information retrieved from the AIRA website in the NanoSerpa v1.0 app, a report like the one shown in Figure 9 is obtained.

The report shows a medium to high health risk (5 points on a scale of 1–10) since a high quantity of black carbon was released to the environment. Emissions obtained for air and soil are of 1.2 and non-existent in the case of water. This latter consideration should be taken with caution since if there were any rivers or lakes in the proximity, they could have been affected by the release of these particles. However, there is not enough information about the event to make a more detailed assessment. A medium to high risk index (7.5 points) was estimated; this fact is consistent with the observations made during the event since housing and landscape were blanketed within a perimeter of several kilometers. Regarding the safety of the workers, it should be noted that although the release of black carbon was large, the event took place in an open space, so the exposure of workers in this case was not relevant.

#### 3.3.4. Fall of Eight TiO_2_ Bulk Bags on the Road

**Figure 10:**
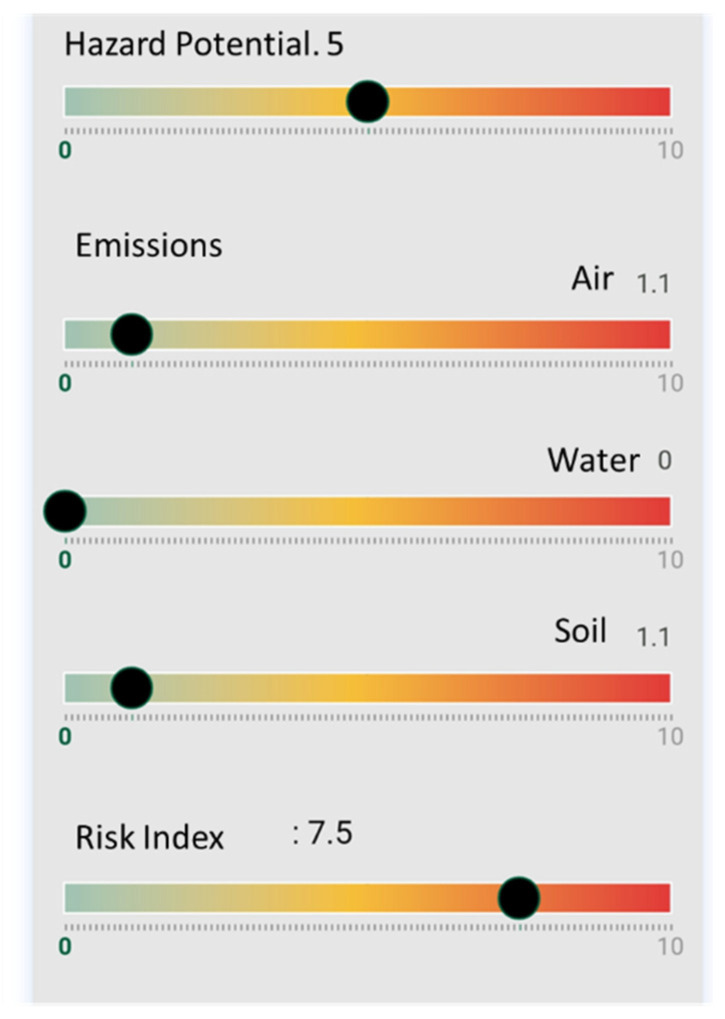
Excerpt from the report generated with the NanoSerpa v1.0 app for a fall of eight TiO_2_ bulk bags on the road. These results apply only to the exposure area where the events studied have occurred.

The fall of eight TiO_2_ bulk bags (total approx. 100 kg) during its transportation on the road was simulated with the NanoSerpa app. The report obtained is shown in Figure 10.

Assuming that some of the bags broke during the fall, an important release of particles should be expected; however, since the event took place in an open space, the levels of particles released into the air are suspected to be low (air emission 1.1 estimated by NanoSerpa app). However, the use of personal protection systems, FFP3 filter mask, gloves, gowns and universal mounted goggles should be considered for workers when collecting spillage and removing fallen bags. In this scenario, a medium health risk could be considered (5 points on the NanoSerpa scale). The soil emission estimated was 1.1. This fact is coherent with the real scenario, since during the event, a few dozen kilograms of material remained unrecovered.

As has been shown in this section, the NanoSerpa app has proven to be useful to the insurance sector. By introducing the few inputs needed (type of nanomaterial, quantity used, etc.), the user can easily obtain a risk index to evaluate the importance of the event in terms of work exposure. Besides, since this app can be installed in smartphones, a quick and easy evaluation of the event can be done, giving the insurance sector the opportunity to streamline procedures and create reports quickly and easily. Moreover, the associated risks for the emissions to the air, water and soil that the NanoSerpa app gives may also be useful as a point of departure to make an environmental impact report associated with the accident or event considered.

## 4. Conclusions

The utility of the NanoSerpa app for nanomaterial risk assessments was tested by simulating different accidents for small- and big-scale scenarios. The risk evaluation obtained seems to be in good agreement with experimental data when they were available. Comparison of the evaluation obtained with the app for real scenarios reported in literature also seems to be consistent.

NanoSerpa v1.0 is an intuitive, user-friendly application that allows workers, technicians and every user to use it without specific training. Besides, inputs needed for this app are not difficult to find, and usually the required information is available in safety data sheets.

Moreover, NanoSerpa v1.0 presents a list of preventive actions that can be applied to minimize or even eliminate the risk of exposure of the worker during a particular accidental release of nanomaterials, and it has proven to be a useful tool for the realization of expert reports in the case of accidents related to nanomaterials. In addition, this application can be used to search and consult the properties of the most commonly used nanomaterials.

The exposure levels in terms of particle number concentrations and size distribution measured by means of direct reading instruments and samplers revealed the presence of particles in the nanometer range in the particle breathing zone during accidental events simulated in a pilot plant, indicating a release of ultrafine particles.

It was observed that the emission levels are directly influenced by the type of handling activity, and not only by the amount used. Hence, to better understand the activities leading to workers’ exposure in the construction sector, an in-depth analysis of the energy involved in the process and the application mode is needed.

## Figures and Tables

**Table 1:** Transfer coefficients.

Flow	TCs (%)
ENPP->Air	5.00
ENPP->Water	6.00
ENPP->Soil	0.01
ENPP->NAMF	88.99
NAMF->Air	10.87
NAMF->Water	7.15
NAMF->Soil	0.58
NAMF->Products	81.40
Products->Air	5.00%
Products->Water	0.00%
Products->Soil	5.00%
Products->WIP	50.00%
Products->STP	5.00%
Products->Export	35.00%
STP->Air	0.00%
STP->Water	3.00%
STP->Soil	0.00%
STP->WIP	97.00%
WIP->Filter	30.00%
WIP->Export	70.00%
Filter->Air	1.00%
Filter->Export	99.00%
Air->Water	3.00%
Air->Soil	97.00%

Note: ENPP: production of engineered nanoparticles; NAMF: nano-article manufacturing, production of articles containing nanoparticles; WIP: waste incineration plant; STP: sludge treatment plant.

**Table 2:** Calculating the risk index based on health hazard and emission.

Emission Hazard	Very Low	Low	Medium	High	Very High
Very low	0	2.5	2.5	5	5
Low	2.5	2.5	5	5	7.5
Medium	2.5	5	5	7.5	7.5
High	5	5	7.5	7.5	10
Very high	5	7.5	7.5	10	10

**Table 3:** Data measured during paint spill containing graphene.

Equipment	Weight Fraction	Time of Exposure (BG/Act/Pers)	Averaged Graphene Content—8 h TWA Workplace	Averaged Graphene Content—8 h TWA Personal	Units	Corrected RCR Graphene (Workplace)	Corrected RCR Graphene (Personal)
CPC 3007 TSI	0.%	10 min	1.53 × 10^0^	2.41 × 10^0^	#/cm^3^	1.63 × 10^−5^	2.57 × 10^−5^
OPS 3330 TSI	0.1%	10 min	1.05 × 10^−2^	1.65 × 10^0^	#/cm^3^	1.12 × 10^−7^	1.76 × 10^−5^
	0.1%	10 min	5.32 × 10^−3^	1.72 × 10^0^	µg/m^3^	1.19 × 10^−4^	3.84 × 10^−2^
CPC 3007 TSI	0.5%	10 min	7.44	8.03 × 10^0^	#/cm^3^	7.94 × 10^−5^	8.57 × 10^−5^
OPS 3330 TSI	0.5%	10 min	6.37 × 10^−2^	4.11 × 10^−2^	#/cm^3^	6.80 × 10^−7^	4.39 × 10^−7^
	0.5%	10 min	2.66 × 10^−2^	3.47 × 10^−2^	µg/m^3^	5.96 × 10^−4^	7.76 × 10^−4^

RCR: risk characterization ratio.

**Table 4:** Input data for NanoSerpa v1.0 simulated scenarios.

	Input Data for NanoSerpa v1.0 Simulated Scenarios
**Group**	Graphene
**Nanomaterial**	Graphene m-GO
**Quantity**	10 mg
**EHP**	High
**State**	Liquid
**Dustiness**	-
**Humidity**	-
**Viscosity**	Low
**Solubility**	Low
**Physical-Chemical Properties**	Thickness: 0.9 nm
**Toxicology**	Size: 430 nm
**Type of Information**	No data
**Measurement**	Direct measurement

**Table 5:** Concentrations of PM10, PM4, PM2.5 and PM1 during the activity and non-activity periods.

	PM1	PM2.5	PM4	PM10	Total
Non-activity (mg/cm^3^)	0.133	0.143	0.180	0.350	0.496
Accidental spillage (mg/cm^3^)	1.210	1.260	1.479	3.041	6.415

**Table 6:** Input data used for NanoSerpa v1.0 simulated scenarios.

	Input Data for NanoSerpa v1.0 Simulated Scenarios
**Group**	TiO_2_
**Nanomaterial**	TiO_2_
**Quantity**	25 kg
**EHP**	Low
**State**	Solid
**Dustiness**	Medium
**Humidity**	Low
**Physical-Chemical Properties**	Particle size: 65 nm
**Toxicology**	No data
**Type of Information**	Documental
**Measurement**	Direct measurement

**Table 7:** Comparison of the risk obtained with the NanoSerpa app and the RCR values obtained from experimental measurements.

Scenario	RCR_EXPERIMENTAL_	NanoSerpa App RISK
Paint spill containing graphene	Workplace: 1.12 × 10^−7^–5.96 × 10^−4^ (<<<1): Very low risk Personal: 4.39 × 10^−7^–3.84 × 10^−2^ (<<<1): Very low risk	Air emissions: 0.9 (very low risk) Risk index: 2.5 (low risk)
Accidental spillage of a dry mortar containing TiO_2_ particles	Workplace: 0.64 (<1) low risk Personal: Not directly evaluated but a higher risk could be expected for the peak concentrations observed during measurements	Air emissions: 1.1 (low risk) Risk index: 7.5 (medium–high risk)

**Table 8:** Input data used for NanoSerpa v1.0-simulated scenarios.

	SiO_2_ Nanoparticle Leak from a Vacuum Cleaner	Release of TiO_2_ from a Bag Filter System	Fall of Eight TiO_2_ Bulk Bags on the Road	Leak of Black Carbon during Transportation
Group	SiO_2_	TiO_2_	TiO_2_	Black carbon
Nanomaterial	SiO_2_	TiO_2_	TiO_2_	Black carbon
Quantity	25 kg	1 kg	100 kg	5 tons
EHP	High	Medium	Low	Medium
State	Solid	Solid	Solid	Solid
Dustiness	High	Medium	Medium	Medium
Humidity	Low	Low	Low	Low
Viscosity	-	-		-
Solubility	-	-		-
Physical-Chemical Properties	*Z*-potential: −25.85 mV			
Toxicology	Specific surface: 200 m^2^/g			
Type of Information	Size: 16 nm	Size: 65 nm	Size: 65 nm	Size: 14 nm
Measurement	Specific surface: 300 m^2^/g			

## Appendix A

**Table A1:** Most cited models to assess exposure and manage risk when dealing with nanomaterials and nano-enabled products.

AVAILABLE TOOLS	Criteria								
	Applicable for Workplace Safety	Applicable for Consumer Risk Assessment	Applicable for Environmental Risk Assessment	Comparison with Risk Control Cost	Quantitative Estimation/Information	Uncertainty Analysis	Life Cycle Perspective	Documented Applications	Assessment Tier
Nanomaterials Control Banding Tool	+	−	−	−	−	−	−	−	Low
NanoSafer	+	−	−	−	+	−	+	+	Low
Advanced REACH Tool (ART)	+	−	−	−	+	−	−	+	High
Precautionary Matrix for Synthetic Nanomaterials	+	+	+	−	−	±	±	−	Low
Tool for ENM-Application Pair Risk Ranking (TEARR)	+	+	−	−	±	−	±	−	Low
Dermal Advanced REACH Tool (DART)	+	+	−	−	+	−	−	−	Low/high
SUN Tiered Occupational and Consumer Exposure Model	+	+	−	−	±	+	−	+	Low/high
LICARA	+	+	+	+	±	−	+	−	Low
SUN DSS	+	+	+	Performs socioeconomic analysis (SEA) to check if the benefits of using certain nanoproducts significantly outweigh their risks	+	+	+	+	Low/high
GUIDEnano DSS	+	+	+	−	+	+	+	−	Low/high

## References

[1] (2011). Impact of Engineered Nanomaterials on Health: Considerations for Benefit Risk Assessment.

[2] Jeevanandam J., Barhoum A., Chan Y.S., Dufresne A., Danquah M.K. (2018). Review on nanoparticles and nanostructured materials: History, sources, toxicity and regulations. Beilstein J. Nanotechnol..

[3] Baalousha M., Cornelis G., Kuhlbusch T., Lynch I., Nickel C., Peijnenburg W., Brink N.V.D. (2016). Modeling nanomaterial fate and uptake in the environment: Current knowledge and future trends. Environ. Sci. Nano.

[4] Gutleb A.C., Cambier S., Fernandes T., Georgantzopoulou A., Kuhlbusch T.A.J., Lynch I., Macken A., Mehennaoui K., Mowller R., Nickel C., Krishnamoorthy S. (2015). Chapter 4: Environmental Fate and Effects of Nanomaterials in Aquatic Freshwater Environments. Nanomaterials—A Guide to Fabrication and Applications.

[5] Keller A.A., McFerran S., Lazareva A., Suh S. (2013). Global life cycle releases of engineered nanomaterials. J. Nanopart. Res..

[6] Baron M. (2015). Safe Handling of Nano Materials and Other Advanced Materials at Workplaces.

[7] John A., Küpper M., Manders-Groot A., Debray B., Lacome J.-M., Kuhlbusch T. (2017). Emissions and Possible Environmental Implication of Engineered Nanomaterials (ENMs) in the Atmosphere. Atmosphere.

[8] Giese B., Klaessig F., Park B., Kaegi R., Steinfeldt M., Wigger H., Von Gleich A., Gottschalk F. (2018). Risks, Release and Concentrations of Engineered Nanomaterial in the Environment. Sci. Rep..

[9] Le Reference du Retour d’Expérience sur Accidents Technologiques. https://www.aria.developpement-durable.gouv.fr/.

[10] Boowook K., Hyunwook K., Il J.Y. (2014). Assessment of Nanoparticle Exposure in Nansilica Handling Process: Including Characteristics of Nanoparticles Leaking from a Vacuum Cleaner. Ind. Health.

[11] Ji J.H., Kim J.B., Lee G., Noh J.-H., Yook S.-J., Cho S.-H., Bae G.-N. (2015). Workplace Exposure to Titanium Dioxide Nanopowder Released from a Bag Filter System. Biomed Res. Int..

[12] NIOSH 2011 (2011). Occupational Exposure to Titanium Dioxide.

[13] NIOSH 2013 (2013). Occupational Exposure to Carbon Nanotubes and Nanofibers.

[14] Zalk D.M., Kamerzell R., Paik S., Kapp J., Harrington D., Swuste P. (2010). Risk Level Based Management System: A Control Banding Model for Occupational Health and Safety Risk Management in a Highly Regulated Environment. Ind. Health.

[15] Stoffen Manager Nano. http://nano.stoffenmanager.nl./.

[16] ISO/TS 12901-2:2014. http://iso.org/.

[17] Hristozov D., Gottardo S., Semenzin E., Oomen A., Bos P., Peijnenburg W., van Tongeren M., Nowack B., Hunt N., Brunelli A. (2016). Frameworks and tools for risk assessment of manufactured nanomaterials. Environ. Int..

[18] West G.H., Lippy B.E., Cooper M.R., Marsick D., Burrelli L.G., Griffin K.N., Segrave A.M. (2016). Toward responsible development and effective risk management of nano-enabled products in the U.S. construction industry. J. Nanopart. Res..

[19] Pietroiusti A., Stockmann-Juvala H., Lucaroni F., Savolainen K. (2018). Nanomaterial exposure, toxicity, and impact on human health. Wiley Interdiscip. Rev. Nanomed. Nanobiotechnol..

[20] Höck J., Hofmann H., Krug H., Lorenz C., Limbach L., Nowack B. (2008). Guidelines on the Precautionary Matrix for Synthetic Nanomaterials.

[21] Höck J., Epprecht T., Furrer E., Hofmann H., Höhner K., Krug H. (2011). Guidelines on the Precautionary Matrix for Synthetic Nanomaterials.

[22] Hansen S.F., Alstrup-Jensen K., Baun A. (2011). NanoRiskCat—A Conceptual Model for Risk Classification of Nanomaterials.

[23] Hansen S.F., Jensen K.A., Baun A. (2013). NanoRiskCat: A conceptual tool for categorization and communication of exposure potentials and hazards of nanomaterials in consumer products. J. Nanopart. Res..

[24] Paik S.Y., Zalk D.M., Swuste P. (2008). Application of a Pilot Control Banding Tool for Risk Level Assessment and Control of Nanoparticle Exposures. Ann. Occup. Hyg..

[25] Kristensen H.V., Hansen S.V., Holm G.R. (2010). Nanopartikler i Arbejdsmiljøet—Viden og Inspiration om Håndtering af Nanomaterialer.

[26] Widler T., Meili C., Semenzin E., Subramanian V., Zabeo A., Hristozov D., Marcomini A., Murphy F., McAlea E.M., Mullins M. (2016). Organisational Risk Management of Nanomaterials Using SUNDS: The Contribution of CENARIOS^®^. Innovation, Technology, and Knowledge Management. Managing Risk in Nanotechnology.

[27] Som C., Zondervan-van den Beuken E., Van Harmelen T., Güttinger J., Bodmer M., Brouwer D., Buist H.E., Carroll R., Coll C., Fransman W. (2014). LICARA Guidelines for the Sustainable Competitiveness of Nanoproducts.

[28] IRGC (2005). Risk Governance: Towards an Integrative Approach. http://www.irgc.org/publications/coreconcepts-of-risk-governance/IRGC(2007)Nanotechnologyrisk.

[29] eNanoMapper Database. https://data.enanomapper.net.

[30] Nanodesk Tools: The Platform. http://sudoenanodesk.net/elearning.

[31] Nowack B. (2017). Evaluation of environmental exposure models for engineered nanomaterials in a regulatory context. NanoImpact.

[32] Monteiro J.V.D., Banerjee S., Ramachandran G. (2011). B2Z: An R Package for Bayesian Two-Zone Models. J. Stat. Softw..

[33] Nicas M. (2000). Mathematical Models for Estimating Occupational Exposure to Chemicals, Chapter Two-Zone Model.

[34] Python. https://www.python.org/.

[35] Schneider T., Brouwer D.H., Koponen I.K., Jensen K.A., Fransman W., Van Duuren-Stuurman B., Van Tongeren M., Tielemans E. (2011). Conceptual model for assessment of inhalation exposure to manufactured nanoparticles. J. Expo. Sci. Environ. Epidemiol..

[36] Tsang M.P., Li D., Garner K.L., Keller A.A., Suh S., Sonnemann G.W. (2017). Modeling human health characterization factors for indoor nanomaterial emissions in life cycle assessment: A case-study of titanium dioxide. Environ. Sci. Nano.

[37] Ganser G.H., Hewett P. (2016). Models for nearly every occasion: Part II—Two box models. J. Occup. Environ. Hyg..

[38] Gottschalk F., Scholz R.W., Nowack B. (2010). Probabilistic material flow modeling for assessing the environmental exposure to compounds: Methodology and an application to engineered nano-TiO_2_ particles. Environ. Model. Softw..

[39] Spinazzè A., Cattaneo A., Borghi F., Del Buono L., Campagnolo D., Rovelli S., Cavallo D.M. (2019). Probabilistic approach for the risk assessment of nanomaterials: A case study for graphene nanoplatelets. Int. J. Hyg. Environ. Health.

[40] Air Quality Index (AQI) Basics. https://www.airnow.gov/aqi/aqi-basics/.

[41] Simple European Calculator of DNEL Tool (SECO) (2012). Characterisation of Dose [Concentration]-Response for Human Health R.8.

[42] ACGIH (2009). TLVs® and BEIs® Based on the Documentation of the Threshold Limit Values for Chemical Substances and Physical Agents and Biological Exposure Indices.

